# Mathematical Analysis and Micro-Spacing Implementation of Acoustic Sensor Based on Bio-Inspired Intermembrane Bridge Structure

**DOI:** 10.3390/s21093168

**Published:** 2021-05-03

**Authors:** Xiang Shen, Liye Zhao, Jiawen Xu, Xuwei Yao

**Affiliations:** 1 Key Laboratory of Micro-Inertial Instrument and Advanced Navigation Technology, Ministry of Education, School of Instrument Science and Engineering, Southeast University, Nanjing 210096, China; xiangshen@seu.edu.cn (X.S.); jiawen.xu@seu.edu.cn (J.X.); wesley.yao@rock-chips.com (X.Y.); 2 Rockchip Electronics Co., Ltd., Fuzhou 350003, China

**Keywords:** *Ormia ochracea*, intermembrane bridge structure, directional recognition of sound source, amplification of phase and amplitude, mathematical analysis and structural simulation, microscale implementation

## Abstract

A biomimetic study on the auditory localization mechanism of *Ormia ochracea* was performed to improve the localization ability of small acoustic systems. We also present a microscale implementation of an acoustic localization device inspired by the auditory organ of the parasitic *O. ochracea*. The device consists of a pair of circular membranes coupled together with an elastic beam. The coupling serves to amplify the difference in magnitude and phase between the two membranes’ responses as the incident angle of the sound changes, allowing directional information to be deduced from the coupled device response. The research results show that the intermembrane bridge structure improves the sound source localization and directional weak acoustic signal acquisition of sound detectors. The recognition rate of the phase difference and amplitude ratio was greatly improved. The theoretical resolution of the incident angle of the sound source can reach 2° at a phase difference recognition rate of 5°. The sound source’s optimal identification frequency range for the coupling device based on the intermembrane bridge bionic structure is 300 Hz to 1500 Hz.

## 1. Introduction

For an animal to localize auditory stimuli, it is typically necessary that both ears be excited by the pressure field [1]. The most important cues available for localization are minute differences in the intensity and time of arrival of the sound between the ear nearest and the ear farthest from the sound source. An animal’s ability to localize sound depends on its ability to detect these minute differences. Many animals have developed mechanisms for effectively increasing these differences before the sound stimulus reaches auditory receptor cells. *Ormia ochracea* (*O. ochracea*) is a parasitoid fly [2,3]. Gravid female flies locate their hosts, male crickets, by homing in on their loud, persistent songs. *O. ochracea* must deal with minimal interaural difference cues to guide directional hearing because of its small body size [4]. The host cricket’s calling song is an amplitude-modulated 5 kHz tone. However, the fly measures less than 1 cm in any aspect, and the distance between its eardrums is approximately 0.5 mm. This means that 5 kHz sound waves are not diffracted by the fly’s body and generate no interaural intensity difference [4]. The interaural time difference is frequency independent and depends only on the speed of sound and the distance between the two ears. The maximal interaural time difference in *O. ochracea* at 90° azimuth is 1.5 μs and decreases to zero for a sound source on the midline axis (0° azimuth). This minuscule interaural time difference is the only physical cue available for the computation of source direction. However, *O. ochracea* can reliably localize cricket songs both in nature and in the laboratory [2,3]. The principal evolutionary innovation responsible for *O. ochracea’s* ability to overcome its unfavorable acoustic physics is a pair of anatomically and functionally coupled eardrums [4,5]. The mechanical resonance of the *O. ochracea* peripheral auditory apparatus in a directional sound field transforms the minuscule time delay in the free field into two cues that its nervous system can use.

**Figure 1:**
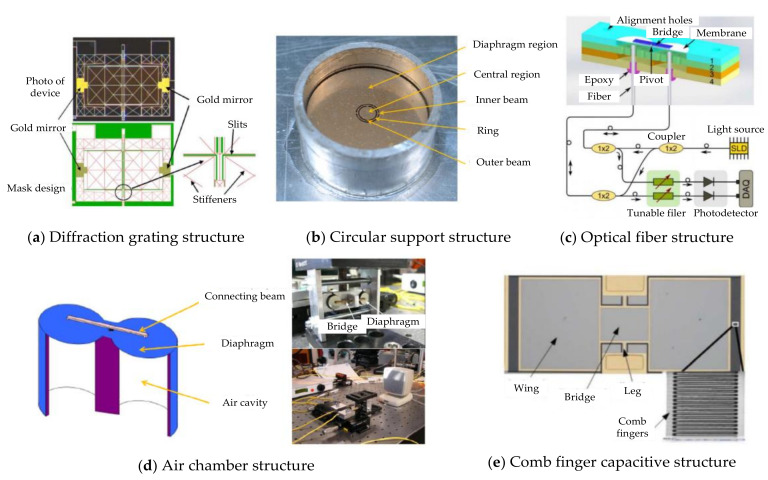
Typical bionic structure design based on the *O. ochracea* ear system. (**a**) Diffractive grating structure [15,16]. (**b**) Circular support structure [9,10]. (**c**) Optical fiber structure [18]. (**d**) Air chamber structure [12]. (**e**) Comb finger structure [7,17].

In 1995, Miles R.N. [5] presented the biomechanics of an *O. ochracea* ear and promoted an engineering perspective of the *O. ochracea* ear system in developing a miniature directional microphone. This discovery attracted many researchers to designing microphones mimicking the ear anatomy of *O. ochracea.* Different diaphragm designs, including rectangular [6,7,8], circular [9,10,11] (Figure 1b), square [12] (Figure 1d), perforated [11,12,13], and gimbal [14] designs, have been fabricated and tested. Readout schemes, including capacitance [11], diffraction grating [15,16] (Figure 1a), comb finger [7,17] (Figure 1e), optical fiber [18] (Figure 1c), and most recently piezoelectric [19,20], have been deployed to monitor diaphragm displacement. Scholars have carried out various designs and research work on miniature bionic acoustic localization structures based on this research work. For example, N. A. Hall [21] proposed a biologically inspired piezoelectric microphone, and D. Wilmott [17] designed a bioinspired miniature direction-finding acoustic sensor. H. Chen [22] and X. Cui [23] proposed sound source localization methods to increase the recognition rate of acoustic sensors. R. H. Downey [24] proposed a structural optimization method to increase the sensitivity of microacoustic sensors. Scholars [25,26,27] have reported the mechanism of piezoelectric microdirectional microphones. D. Calero [28] researched some hearing devices with implantable sensors, including different transduction mechanisms (e.g., capacitive, piezoelectric, electromagnetic). Y. Zhang [29] reported the microfabrication processes for insect-inspired acoustic microsensors. N. Kishor Bhaskarrao [30] carried out the analysis of a linearizing direct digitizer with phase-error compensation. This device allows all of the normal membrane modes to operate, including the critical fundamental rocking and bending modes. The acoustic target recognition method [31,32] has also been proposed to improve the recognition rate.

Any pressure-sensitive microphone with an output that depends on the direction of a propagating sound wave must detect the difference in acoustic pressure at a minimum of two points in space. In contrast, directional microphones have been used in audio applications for many decades. They can be very challenging to design for applications that have substantial size constraints. Previous scholars have done preliminary research on the bionic coupling mechanism of the intermembrane bridge structure. A preliminary coupled mathematical model of this bionic structure was also established. However, few reports on the principle of bionic coupling amplification and the microscale implementation of acoustic sensors based on the bio-inspired intermembrane bridge structure. In addition, if the space of the sound source is so close, even it is less than 1/2 wavelength, the phenomenon of acoustic diffraction produces. To solve the problem, we designed a miniature differential microsensor that can accurately detect pressure differences base on the intermembrane bridge bionic structure of *O. ochracea*. The setting of the coupling structure in this paper increases the equivalent spacing of the sensitive unit. The phase difference and amplitude ratio are magnified, improving the recognition rate of the sound source direction. This sensor is suitable for distinguishing the direction of sound sources with low and medium-frequency.

The paper is organized as follows: In Section 2, a mathematical analysis of the intermembrane bridge bionic structure is given. In Section 3, the simulation analysis of the intermembrane bridge bionic structure is executed. Microscale implementation of the intermembrane bridge bionic structure and experimental results are reported and discussed in Section 4. Finally, a set of conclusions are presented in Section 5.

## 2. Mathematical Analysis of the Intermembrane Bridge Bionic Structure

### 2.1. Establishment of the Mechanical Dynamics Model

**Figure 2:**
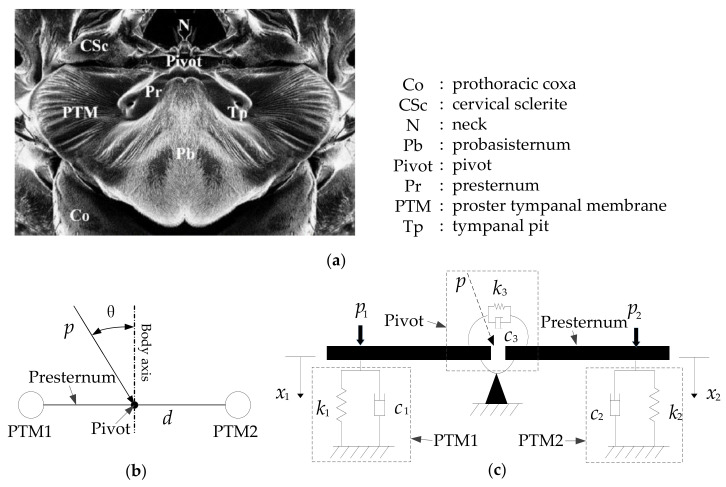
External anatomy and equivalent 2-DOF model and parameters of *O. ochracea’s* ears. (**a**) External anatomy of the ears of *O. ochracea* [5]. (**b**) The relationship between the incident angle of the sound source and the body axis of *O. ochracea*. (**c**) Mechanical model of the ears of *O. ochracea* by Miles R.N. [5].

In the literature [5], a 2-DOF (degrees of freedom) mechanics model has been developed for the *O. ochracea* ear structure, which can help explain the exceptional directional hearing ability of *O. ochracea.* A schematic diagram of the equivalent 2-DOF model and the *O. ochracea* ear parameters is shown in Figure 2. The 2-DOF intermembrane bridge structure is modeled as an elastic and damping structure with boundary conditions. Among them, PTMs and pivots are modeled as spring structures with elastic and damping characteristics. The presternum is modeled as the rigid beam structure. The time-domain characteristics or magnitude characteristics can be changed based on this coupling structure.

Parameters listed in Figure 2 mean: $k_{1}$ and $k_{2}$: translation spring; $c_{1}$ and $c_{2}$: translation dashpot; $k_{3}$: torsional spring; $c_{3}$: torsional dashpot; p: sound wave pressure at the pivot point; θ: The angle of the incident direction of the sound wave and the body axis of *O. ochracea*; $p_{1}$ and $p_{2}$: The left and right sound pressure signals; $x_{1}$ and $x_{2}$: The left and right sound pressure signals; d: The distance between two tympanic membranes.

The equations of motion can be readily obtained as follows [5]:

(1) $$M\left\{\begin{array}{c} \ddot{x_{1}}\\ \ddot{x_{2}} \end{array}\right\}+C\left\{\begin{array}{c} \dot{x_{1}}\\ \dot{x_{2}} \end{array}\right\}+K\left\{\begin{array}{c} x_{1}\\ x_{2} \end{array}\right\}=\left\{\begin{array}{c} p_{1}\\ p_{2} \end{array}\right\}$$

where M is the mass matrix, C is the damping matrix, K is the stiffness matrix. and denote the first- and second-order differentiation concerning time τ. The remaining parameters are consistent with the above.

According to Equation (1), it can be obtained:

(2) $$\left[\begin{array}{cc} m & 0\\ 0 & m \end{array}\right]\left[\begin{array}{c} \ddot{x_{1}}\\ \ddot{x_{2}} \end{array}\right]+\left[\begin{array}{cc} c_{1}{+c}_{3} & c_{3}\\ c_{3} & c_{2}{+c}_{3} \end{array}\right]\left[\begin{array}{c} \dot{x_{1}}\\ \dot{x_{2}} \end{array}\right]+\left[\begin{array}{cc} k_{1}{+k}_{3} & k_{3}\\ k_{3} & k_{2}{+k}_{3} \end{array}\right]\left[\begin{array}{c} x_{1}\\ x_{2} \end{array}\right]=\left[\begin{array}{c} p_{1}\\ p_{2} \end{array}\right]=\left[\begin{array}{c} p.s{.e}^{\frac{\omega \tau }{2}i}\\ p.s{.e}^{-\frac{\omega \tau }{2}i} \end{array}\right]$$

where m is the mass of the bar; s is the area of the unilateral tympanic membrane; ω is the vibration frequency of the sound source; τ is the difference in the sound path between the tympanic membranes on both sides, and τ=dsinθ/v; d is the distance between two tympanic membranes; v is the propagation speed of the sound wave. The remaining parameters are consistent with the above.

Therefore, the *O. ochracea* coupling connection structure can be regarded as a two-input and two-output system. The coupling parameters of the bionic structure determine the nonlinearity characteristics. Miles R.N. [5] has done preliminary research on the bionic coupling mechanism of the intermembrane bridge structure. However, the principle of bionic coupling amplification and the microscale implementation of acoustic sensors based on bio-inspired intermembrane bridge structure has not been studied. The modal analysis of the mathematical model is not carried out. To interpret how the two tympanic membranes interact, we need to solve Equation (2). This is explained in Section 2.2.

### 2.2. Modal Analysis of the Mechanical Dynamics Model

The mechanical model of the *O. ochracea* auditory system qualitatively describes the amplification mechanism of the small difference between the acoustic excitation of the intermembrane bridge structure. In this section, the modal decomposition method is used to research the mechanical model of the intermembrane bridge structure and its dynamic characteristics to determine the location method of the intermembrane bridge sensing structure.

Let $m_{1}{=m}_{2}=m$, $k_{1}{=k}_{2}=k$, $c_{1}{=c}_{2}=c$. For free vibrations, the natural frequencies and mode shapes of the system can be found as:

For free vibrations, the natural frequencies and mode shapes of the system can be found as [33]:

(3) $$\omega_{1}=\sqrt{k/m}{,\omega }_{2}=\sqrt{\left({k+2k}_{3}\right)/m}$$

In the first mode, the two diaphragms are in a rocking state. They are changed to bending mode in the second state. Therefore, the *O. ochracea* auditory system can be regarded as a two-input and two-output system. Two-outputs are sound pressure signals acting on the two tympanic membranes. Parameters $k_{3}$ and $c_{3}$ make the two tympanic membranes no longer independent. They are coupled with each other. It is necessary to solve the second-order differential Equation (2) to determine how the two tympanic membranes interact.

The corresponding displacements at the tympanic membrane expressed as follows:

(4) $$H_{x1p}=\frac{s\left(k_{3}{+i\omega c}_{3}\right)\times \left(e^{\frac{i\omega \tau }{2}}{-e}^{-\frac{i\omega \tau }{2}}\right)+s\left({k+i\omega c-m\omega }^{2}\right)e^{\frac{i\omega \tau }{2}}}{{\left({k+i\omega c+k}_{3}{+i\omega c}_{3}{-m\omega }^{2}\right)}^{2}-{\left(k_{3}{+i\omega c}_{3}\right)}^{2}}$$

(5) $$H_{x2p}=\frac{s\left(k_{3}{+i\omega c}_{3}\right)\times \left(e^{\frac{i\omega \tau }{2}}{-e}^{-\frac{i\omega \tau }{2}}\right)+s\left({k+i\omega c-m\omega }^{2}\right)e^{-\frac{i\omega \tau }{2}}}{{\left({k+i\omega c+k}_{3}{+i\omega c}_{3}{-m\omega }^{2}\right)}^{2}-{\left(k_{3}{+i\omega c}_{3}\right)}^{2}}$$

where $H_{x1p}$ and $H_{x2p}$ represent the displacement responses on the first side and the second side, respectively.

Let $H_{x1\;\times \;2}{=H}_{x1p}{/H}_{x2p}$, Equation (6) is obtained from Equations (4) and (5):

(6) $$H_{x1\;\times \;2}=\frac{s\left(k_{3}{+i\omega c}_{3}\right)\times \left(e^{\frac{i\omega \tau }{2}}{-e}^{-\frac{i\omega \tau }{2}}\right)+s\left({k+i\omega c-m\omega }^{2}\right)e^{\frac{i\omega \tau }{2}}}{s\left(k_{3}{+i\omega c}_{3}\right)\times \left(e^{\frac{i\omega \tau }{2}}{-e}^{-\frac{i\omega \tau }{2}}\right)+s\left({k+i\omega c-m\omega }^{2}\right)e^{-\frac{i\omega \tau }{2}}}$$

The physical coordinates are transformed into modal coordinates, and the two coupled differential equations are transformed into two independent differential equations for solving. The modal pattern of the *O. ochracea* auditory system is:

(7) $$\Phi =\frac{1}{\sqrt{2m}}\left[\begin{array}{cc} 1 & 1\\ -1 & 1 \end{array}\right]$$

The modal array has orthogonality for the mass matrix, stiffness matrix, and damping matrix. So:

(8) $$\left\{\begin{array}{c} {I=\Phi }^{T}M\Phi \\ \mathrm{diag}\left(\omega_{i}^{2}\right){=\Phi }^{T}K\Phi \\ \mathrm{diag}\left({2\xi }_{i}\omega_{i}\right){=\Phi }^{T}C\Phi \end{array}\right.$$

where Φ represents the modal array. I is the unit matrix. $\omega_{i}$ and $\xi_{i}$ are the undamped free oscillation frequency and damping ratio of the $i^{\mathrm{th}}$ mode, respectively. The free oscillation frequency and damping ratio of the first-order and second-order modes are obtained according to Equations (7) and (8):

(9) $$\left\{\begin{array}{c} \omega_{1}=\sqrt{k/m}\\ \omega_{2}=\sqrt{\left({k+2k}_{3}\right)/m}\\ \xi_{1}{=c/(2\omega }_{1}m)\\ \xi_{2}{=(c+2c}_{3}{)/(2\omega }_{2}m) \end{array}\right.$$

According to Equation (9), the oscillation frequency and damping ratio are determined by the system’s mechanical parameters. The coordinate transformation was executed for physical coordinates using a modal array:

(10) x =Φη

where ${\eta =[\eta }_{1}{,\eta }_{2}{]}^{T}$ is the vector of modal coordinate. Psin(ωt) is the sonic excitation acting at the pivot center of *O. ochracea*. The pressure signal acting at the tympanic membranes can be defined as $f_{1}\left(t)=P\sin(\omega t+\omega \tau /2\right)$ and $f_{2}\left(t)=P\sin(\omega t-\omega \tau /2\right)$. Therefore, the modal equation of the sound pressure signal can be expressed as:

(11) $$\left\{\begin{array}{c} \ddot{\eta_{1}}{+2\xi }_{1}\omega_{1}\dot{\eta_{1}}{+\omega }_{1}^{2}\eta_{1}=P\sqrt{2/m}\cos(\omega t)\sin(2\omega \tau /2)\\ \ddot{\eta_{2}}{+2\xi }_{2}\omega_{2}\dot{\eta_{2}}{+\omega }_{2}^{2}\eta_{2}=P\sqrt{2/m}\cos(\omega t)\sin(2\omega \tau /2) \end{array}\right.$$

**Figure 3:**
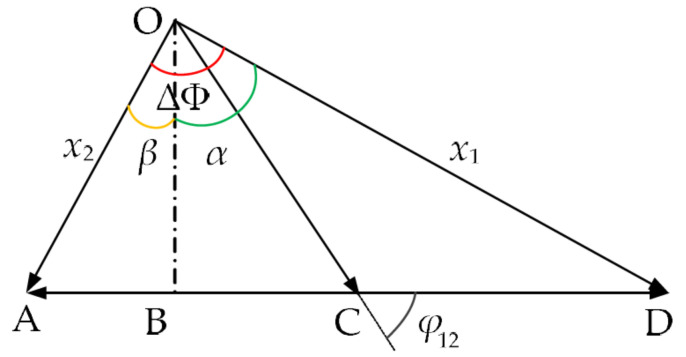
The vector synthesis of the two-order modal response (OB is perpendicular to AD, ΔΦ=α+β).

Modal parameters were obtained by the undetermined coefficient method:

(12) $$\left\{ \begin{array}{l} \eta_{1}{(t)=A}_{1}{\cos(\omega t+\varphi }_{1})\\ \eta_{2}{(t)=A}_{2}{\cos(\omega t+\varphi }_{2})\\ A_{1}=\frac{P\sqrt{2/\mathrm{msin}}\left(\omega \tau /2\right)}{\sqrt{\left(\omega_{1}^{2}{-\omega }^{2}\right){+(2\omega }_{1}\xi_{1}{\omega )}^{2}}}\\ A_{2}=\frac{P\sqrt{2/\mathrm{mcos}}\left(\omega \tau /2\right)}{\sqrt{\left(\omega_{2}^{2}{-\omega }^{2}\right){+(2\omega }_{2}\xi_{2}{\omega )}^{2}}}\\ \varphi_{1}=-\arctan\left(\frac{{2\omega }_{1}\xi_{1}\omega }{\omega_{1}^{2}{-\omega }^{2}}\right)\\ \varphi_{2}=-\arctan\left(\frac{{2\omega }_{1}\xi_{1}\omega }{\omega_{2}^{2}{-\omega }^{2}}\right) \end{array}\right.$$

where $A_{1}$ and $A_{2}$ are the amplitudes of the first-order and second-order modes, respectively. $\varphi_{1}$ and $\varphi_{2}$ are the phases of the first-order and second-order modes, respectively. The system parameters decide the amplitudes and phases of modal responses. Additionally, the frequency of the incident wave also affects the phase of the response. System response in physical coordinates is obtained:

(13) $$\left\{\begin{array}{c} x_{1}{(t)=(A}_{2}\sin\left({\omega t+\varphi }_{2}\right){+A}_{1}\cos\left({\omega t+\varphi }_{1}\right))/\sqrt{2m}\\ x_{2}{(t)=(A}_{2}\sin\left({\omega t+\varphi }_{2}\right){-A}_{1}\cos\left({\omega t+\varphi }_{1}\right))/\sqrt{2m} \end{array}\right.$$

where $x_{1}\left(t\right)$ and $x_{2}\left(t\right)$ are the displacements of two tympanic membranes of *O. ochracea* under the action of acoustic excitation. The tympanic membrane response is the synthesis of first-order and second-order modal responses. Taking the phase of the first-order modal response as a reference, the schematic diagram of the two-order modal response synthesis is shown in Figure 3. In Figure 3, points O, A–C, and D are set in specific locations, and OB is perpendicular to AD.

where ΔΦ is the phase difference between two tympanic membranes. $\varphi_{12}$ is the phase difference between the two-order modes. Let ∠AOB=β, ∠BOD=α. It can be obtained that ΔΦ=α+β. R_1_ and R_2_ represent the amplitudes of $x_{1}\left(t\right)$ and $x_{2}\left(t\right)$, respectively. Amplitude and phase differences of the two tympanic membrane responses can be calculated in Figure 3 and are shown as follows:

(14) $$\left\{ \begin{array}{l} R_{1}=\sqrt{{{(A}_{2}{\sin\varphi }_{12})}^{2}{+(A}_{1}{+A}_{2}{\cos\varphi }_{12}{)}^{2}}\\ R_{2}=\sqrt{{{(A}_{2}{\sin\varphi }_{12})}^{2}{+(A}_{1}{-A}_{2}{\cos\varphi }_{12}{)}^{2}}\\ \tan\left(\alpha \right)=\frac{A_{1}{+A}_{2}{\cos\varphi }_{12}}{A_{2}{\cos\varphi }_{12}}\\ \tan\left(\beta \right)=\frac{A_{1}{-A}_{2}{\cos\varphi }_{12}}{A_{2}{\sin\varphi }_{12}}\\ \tan\left(\Delta \varphi \right)=\frac{\tan\left(\alpha \right)+\tan(\beta )}{1-\tan(\alpha )\tan(\beta )}=\frac{{2A}_{1}A_{2}{\sin\varphi }_{12}}{A_{2}^{2}{-A}_{1}^{2}} \end{array}\right.$$

As seen from Equation (14), the amplitude difference and phase difference of the two tympanic membranes change with the change in the incident angle. Sound source localization can be implemented according to this theory. The setting of the coupling parameters can effectively realize the amplification of the phase difference and the amplitude difference, thereby improving the recognition rate of the sound source direction.

## 3. Simulation Analysis of the Intermembrane Bridge Bionic Structure

### 3.1. Mathematical Simulation Analysis

Simulation analysis was implemented based on the mechanical dynamics model of the bionic membrane bridge structure by MATLAB. The effects of those mechanical parameters on the vibration characteristics of the tympanic membrane on both sides were investigated according to reference [5]. The coupling parameters are set as follows: k=0.576 N/m, $k_{3}=5.18\;N/m$, $m=2.88{\times 10}^{-10}\;\mathrm{kg}$, $c=1.15{\times 10}^{-5}\;N\cdot s/m$, $c_{3\;}=2.88{\times 10}^{-5}\;N\cdot s/m$. The incident frequency is set to 4 kHz. The incident angle is set at 45°.

**Figure 4:**
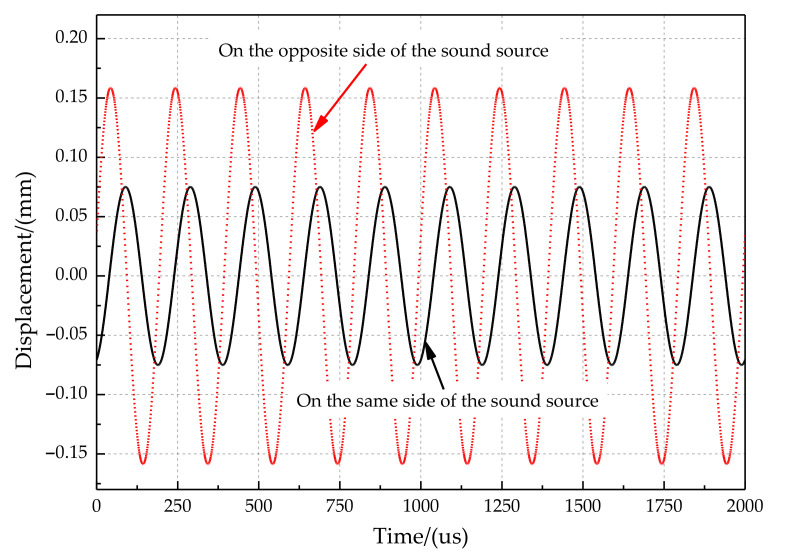
The time-domain response of two tympanic membranes.

The time-domain responses of the two tympanic membranes are shown in Figure 4. Figure 4 shows that the vibration amplitude of the tympanic membrane on the same side of the sound source is more significant than that on the opposite side. The response of the two tympanic membranes has a significant amplitude and time difference under the action of acoustic excitation. Moreover, once the vibration starts, the difference immediately appears. The difference is constant when the incident angle and frequency of the sound wave are constant.

**Figure 5:**
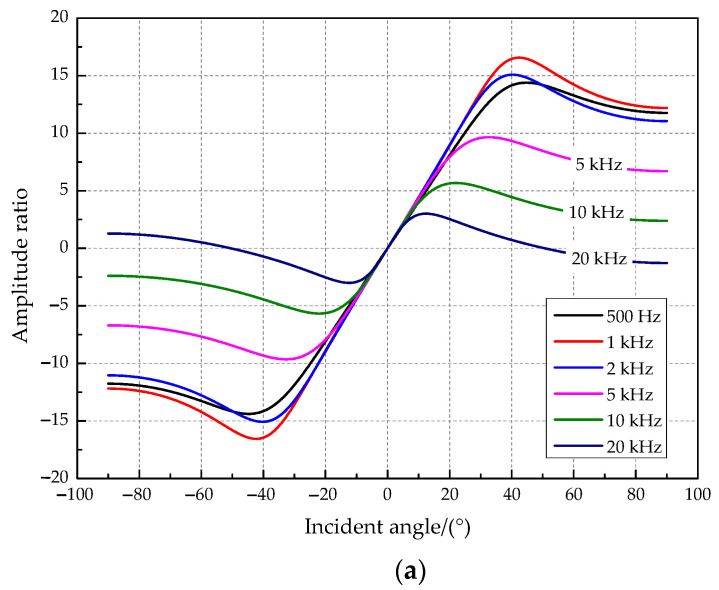

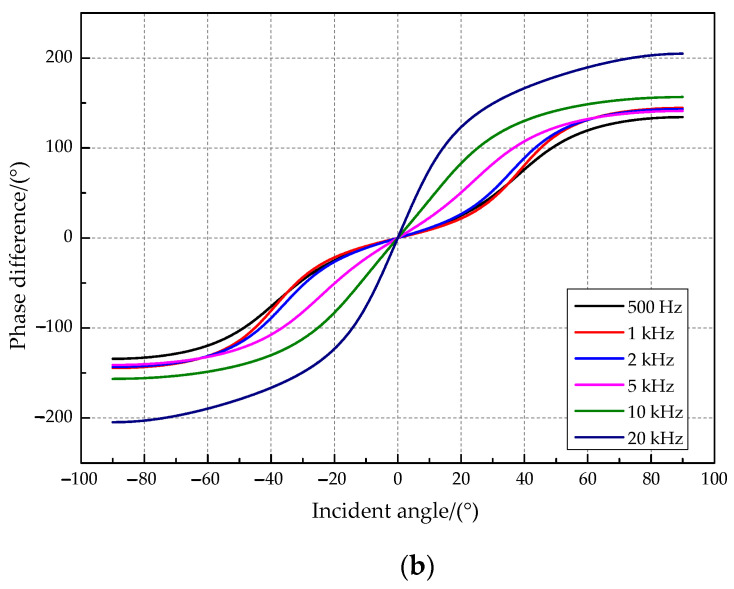
The relationship between key parameters and the incident angle. (**a**) The amplitude ratio and the incident angle. (**b**) The phase difference and the incident angle.

The amplitude difference of the vibration displacement response on both sides of two tympanic membranes was simulated in this paper when the incident frequency was set at 500 Hz, 1 kHz, 2 kHz, 5 kHz, 10 kHz, and 20 kHz. The simulation results are presented in Figure 5. The relationship curves between the amplitude difference and the incident angle (Figure 5a) show that the coupling structure amplifies the amplitude and time difference of the two tympanic membrane responses. The amplitude ratio (“R” _“1”/“R” _“2”) increases and then decreases with the increasing incident angle of the sound source. A sound source incident angle maximizes the amplitude amplification ratio for a sound source signal of a specific frequency. It has a relatively good amplitude amplification effect when the sound source frequency is 1000 Hz, and the incident angle of the sound source is approximately 43°. In addition, the above two parameters have a one-to-one relationship with the incident angle. Therefore, *O. ochracea* can locate the sound source precisely. The relationship curves between the phase difference and the incident angle (Figure 5b) show that the phase difference increases with the angle of incidence. It tends to produce a higher phase magnification for higher source frequencies.

**Figure 6:**
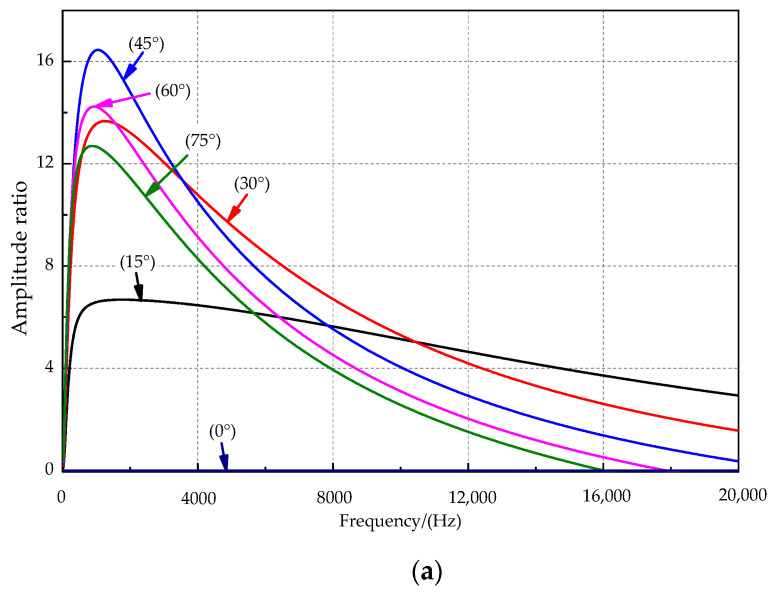

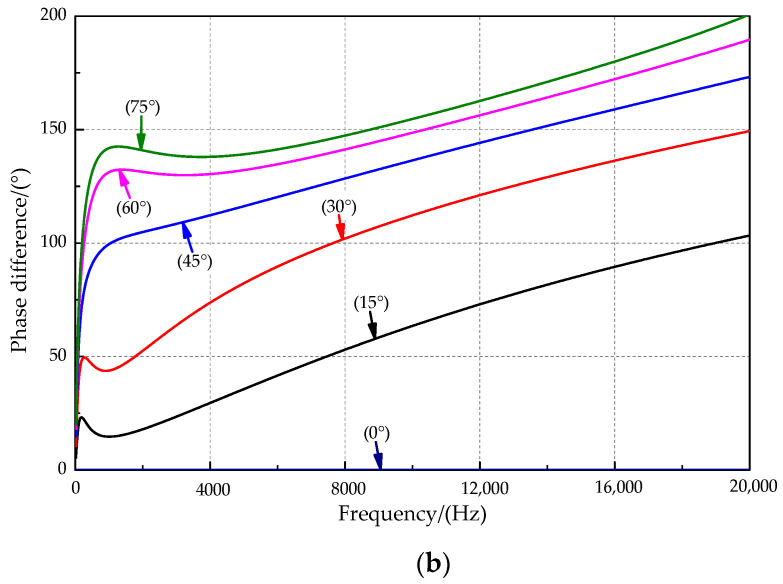
The relationship between key parameters and the frequency. (**a**) The amplitude ratio and the frequency. (**b**) The phase difference and the frequency.

The amplitude and phase difference curves at incident angles of 0°, 15°, 30°, 45°, 60°, and 75° are shown in Figure 6a,b. For the sound source signal frequency of 0–2 kHz, the amplitude ratio rises first and then slowly decreases with increasing incident frequency. The amplitude ratio at the sound source incident angle of 45° is the best among the incident mentioned above angles of sound sources and reaches a maximum of approximately 1000 Hz. The sensitive frequency bands were inconsistent for the amplitude difference and phase difference. Figure 6b shows that the response phase difference appears to increase rapidly with increasing frequency when a sound source of lower frequency is used. The response phase difference tends to increase slowly when the sound source exceeds approximately 1000 Hz, and there is a local maximum phase difference near the sound source at approximately 1000 Hz. As the sound source’s incident angle increases, the local maximum point slowly moves toward the high-frequency direction, and the phase response amplification effect is better.

**Figure 7:**
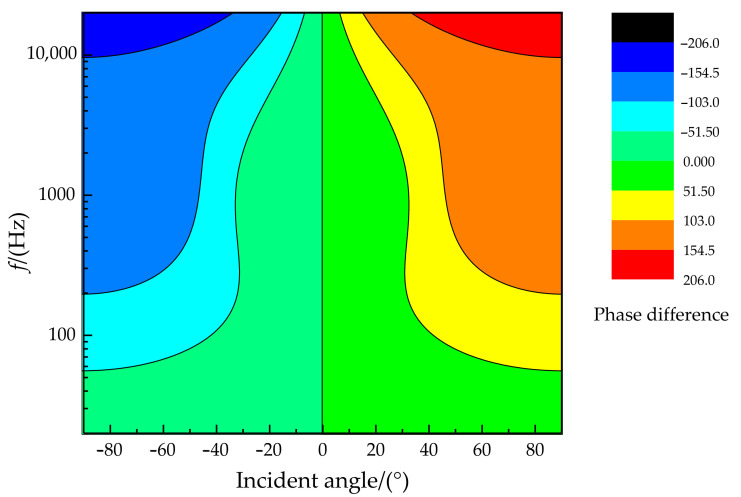
The relationship diagram of phase difference, incident angle, and frequency.

**Figure 8:**
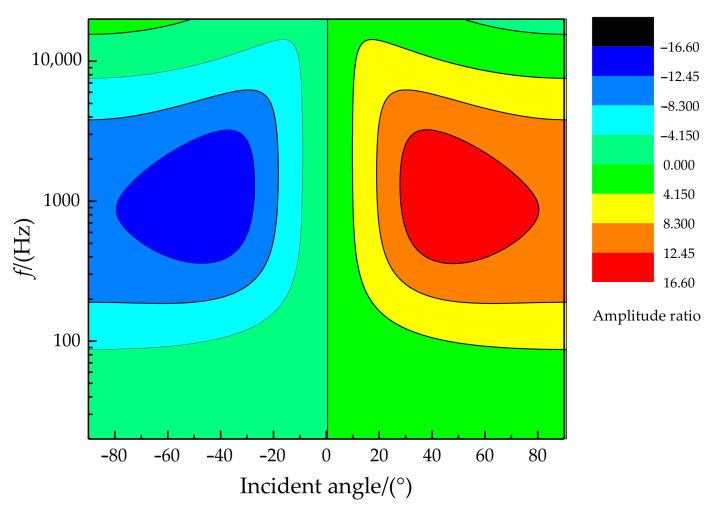
The relationship diagram of amplitude difference, incident angle, and frequency.

The relationship of the phase difference, incident angle, and frequency is illustrated in Figure 7. It can be found that better phase difference sensitivity is presented for higher sound frequencies (in the sound frequency range of 20 Hz~20 kHz). In particular, the phase difference can reach more than 150° when the frequency is greater than 1 kHz at an incident angle of more than 45°. The relationship of the amplitude difference, incident angle, and frequency is shown in Figure 8. There is a specific sensitive frequency range for a type of coupling parameter of the membrane bridge. A significant amplitude difference was presented for a membrane bridge in the sensitive frequency range and the incident angle of the sound source.

### 3.2. Structural Simulation Analysis

**Figure 9:**
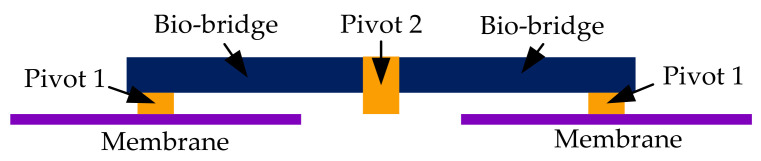
The structure diagram of the established intermembrane bridge structure.

Structural analysis was performed on the established bioinspired intermembrane bridge structure by ANSYS. The incident angle of the sound source is 45°, and the sound pressure is 0.2 Pa (80 dB). The distance between the centers of the two membranes is 10 mm. The structure diagram of the established intermembrane bridge structure is shown in Figure 9. Other structural parameters of the established bioinspired intermembrane bridge structure are listed in Table 1.

**Figure 10:**
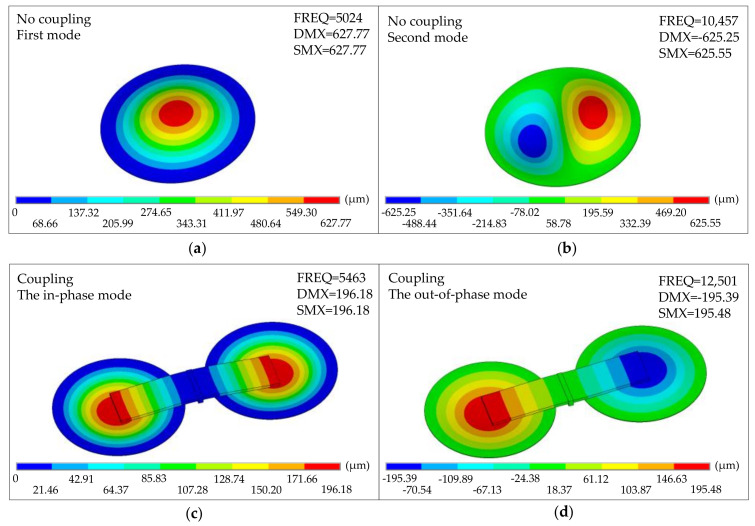
The modal characteristics of the bio-inspired structure. (**a**) The first mode of the non-coupling structure. (**b**) The second mode of the non-coupling structure. (**c**) The in-phase mode of the coupling structure. (**d**) The out-of-phase mode of the coupling structure.

Assuming an equivalent modulus of 4000 MPa (which is an equivalent modulus of the polyester membrane), an uncoupled single membrane structure was analyzed, and the in-phase mode and the out-of-phase mode of the non-coupling structure were observed at 5024 Hz and 10,457 Hz (Figure 10a,b). These two modes show good agreement with finite element model predictions in shape and frequency. For coupled structures, the in-phase mode (dominant bending state) with both membranes operating in phase is approximately 5463 Hz (Figure 10c). It is slightly higher than the uncoupled single membrane, making sense since the beam’s bending adds additional stiffness to the device. The out-of-phase mode is a rocking state, with the two membranes operating in their fundamental mode but 180° out of phase (Figure 10d).

Therefore, simulation results show that the time difference and amplitude difference at different incident directions and different incident frequencies have apparent changes. *O. ochracea* utilizes the membrane bridge’s mechanical coupling structure to amplify the time difference and amplitude difference of the weak signals collected by the tympanic membranes on both sides. *O. ochracea* amplifies a weak sound signal relying on this nonlinear intermembrane bridge coupling structure to localize the host sound source.

## 4. Microscale Implementation of the Intermembrane Bridge Bionic Structure

### 4.1. Design and Fabrication

**Figure 11:**
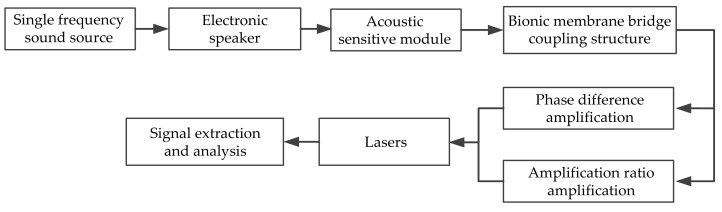
The experimental schematic in this paper.

The sound source positioning experiment was carried out based on the self-built laser detection system of the intermembrane bridge bionic structure in this paper. It was developed in an anechoic room to reduce the interference of environmental noise. The experimental schematic is illustrated in Figure 11. First, multiple sound source signals of some fixed frequency were generated by MATLAB. Then, they were played by an audio player and magnified by a loudspeaker. The acoustic-sensitive module was used to sense the sound signal and convert it into a vibration signal of the membrane. The vibration signals were amplified under the intermembrane bridge coupling structure, collected and analyzed by the laser signal extraction system.

**Figure 12:**
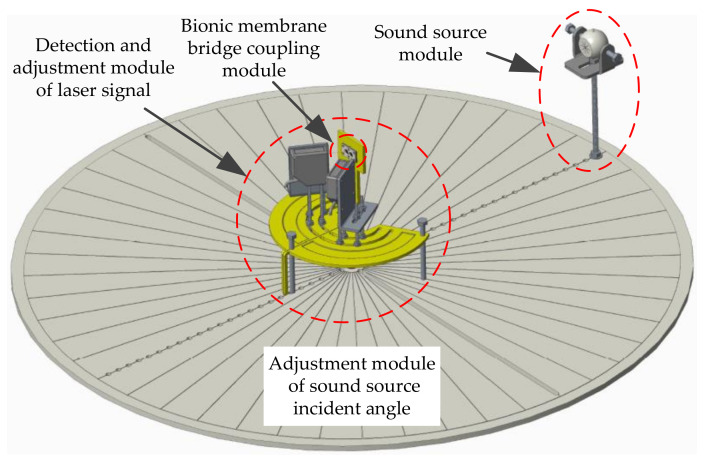
The laser detection system for sound source localization.

The laser detection system for sound source localization includes four modules (Figure 12): the intermembrane bridge bionic acoustic-sensitive module, the detection and adjustment module of the laser signal, the sound source module, and the adjustment module of the sound source incident angle. The sound source incident angle is regulated by rotating the dial. When the sound source is incident at the angle of θ, two acoustic-sensitive membranes are excited. The vibration signals of the acoustic-sensitive membrane were calculated by laser detection. The incident angle α of the lasers was adjusted by the laser position adjustment plate. Therefore, *x* = *x*′*cosα*, where *x* is the vibration amplitude of the acoustic-sensitive membrane and *x*′ is the vibration amplitude in the direction of the laser light path.

**Figure 13:**
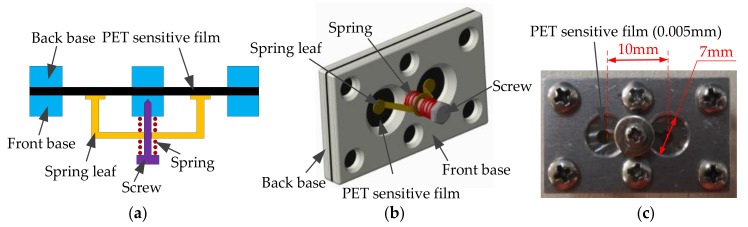
The acoustic-sensitive module based on the inter-membrane bridge bionic structure. (**a**) Plane structure. (**b**) 3D structure. (**c**) Physical photo.

The acoustic-sensitive module based on the intermembrane bridge bionic structure is shown in Figure 13. It includes acoustic-sensitive membranes, front bases, rear bases, and intermembrane bridge spring assemblies. The material of the membrane is a chrome-plated polyester (PET)-sensitive film, and its thickness is 0.005 mm. The distance between the centers of the two membranes is 10 mm, and the diameter of the membrane is 7 mm. The spring piece made of copper–zinc alloy is bonded to the acoustic-sensitive membrane and is fixed to the helical spring.

**Figure 14:**
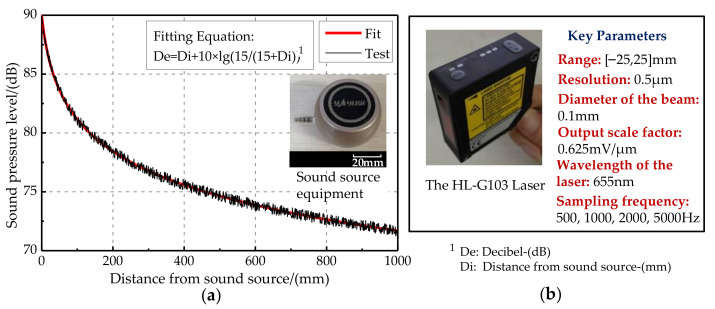
Characteristics of key equipment. (**a**) Characteristics of the sound source equipment. (**b**) Characteristics of the laser.

**Figure 15:**
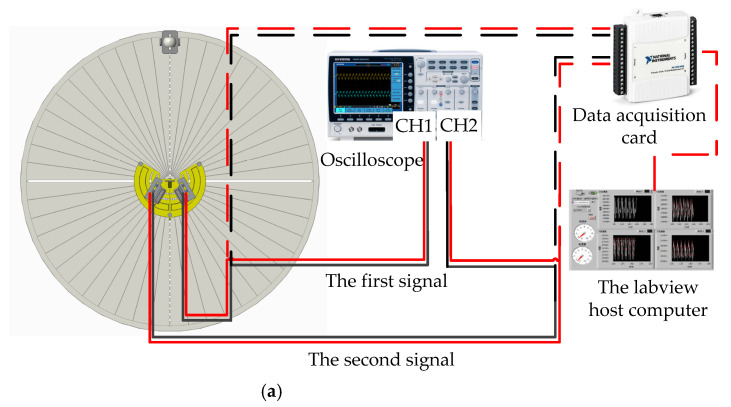

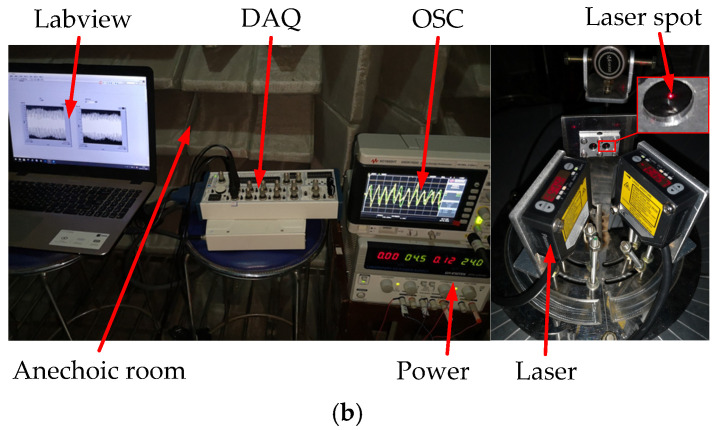
The signal acquisition process of the test experimental. (**a**) The signal acquisition scheme of the test experiment. (**b**) The experimental photos.

The test experiment was carried out in an anechoic room (which can effectively reduce the interference of echo and external noise). The sound source equipment was selected and tested first (as shown in Figure 14a). When the output loudness of the sound source equipment is 90 dB, the relationship of the decibel and distance is De = Di + 10 lg(15/(15 + Di)). The error standard deviation of the loudspeaker sound pressure level is 0.053 dB, meeting the experimental requirements. The signal is detected by the laser and collected by the signal acquisition card. The HL-G103 standard laser sensors were selected for this experiment. The key parameters of the laser sensor are list in Figure 14b. Their displacement resolution was set at 0.5 μm, and sampling frequency was set at 5 kHz. Moreover, the displacement sensitive range of the laser sensors was set as [−25,25] μm, and the output scale factor was set at 0.625 mV/μm. The incident angle of the laser light path relative to the vertical bisecting plane of the two vibrating membranes was ±30°. The irradiation point of the laser sensor was at the center of the vibrating membrane. The center distance between the laser and the vibrating membrane was approximately 30 mm. The vibration signals converted by the laser sensors were collected by a data acquisition card. Then, they were displayed by an oscilloscope (OSC). The signal acquisition scheme and experimental photos are shown in Figure 15a,b, respectively. Simultaneously, lasers were connected to the NI-USB 6251 data acquisition (DAQ) board and displayed through the LABVIEW host computer in real time. The sampling rate of the DAQ board was set as 10 kHz.

### 4.2. Experiment Analysis of Acoustic Signal Characteristics

#### 4.2.1. Analysis of the Sound Source Incident Angle Characteristic

To explore the amplification effect of the intermembrane bridge bionic device, the angle and frequency of the sound source for the bionic coupling structure and uncoupling structure are studied separately in this test experiment. First, the non-coupling structure and the intermembrane bridge bionic structure were tested under different sound source incident angles to explore the acoustic sensitivity characteristics. The acoustic sensitivity characteristics of the incident angle of [0°, 90°] and the step size of 5° were tested for the bionic coupling structure. The other conditions of the experiment are set as follows:

- The frequency of the sound source was 500 Hz;
- The distance between the front surface of the speaker and the midpoint of the two diaphragms was 100 mm;
- The sound pressure level of the sound source on the front surface of the speaker was 90 dB.

**Figure 16:**
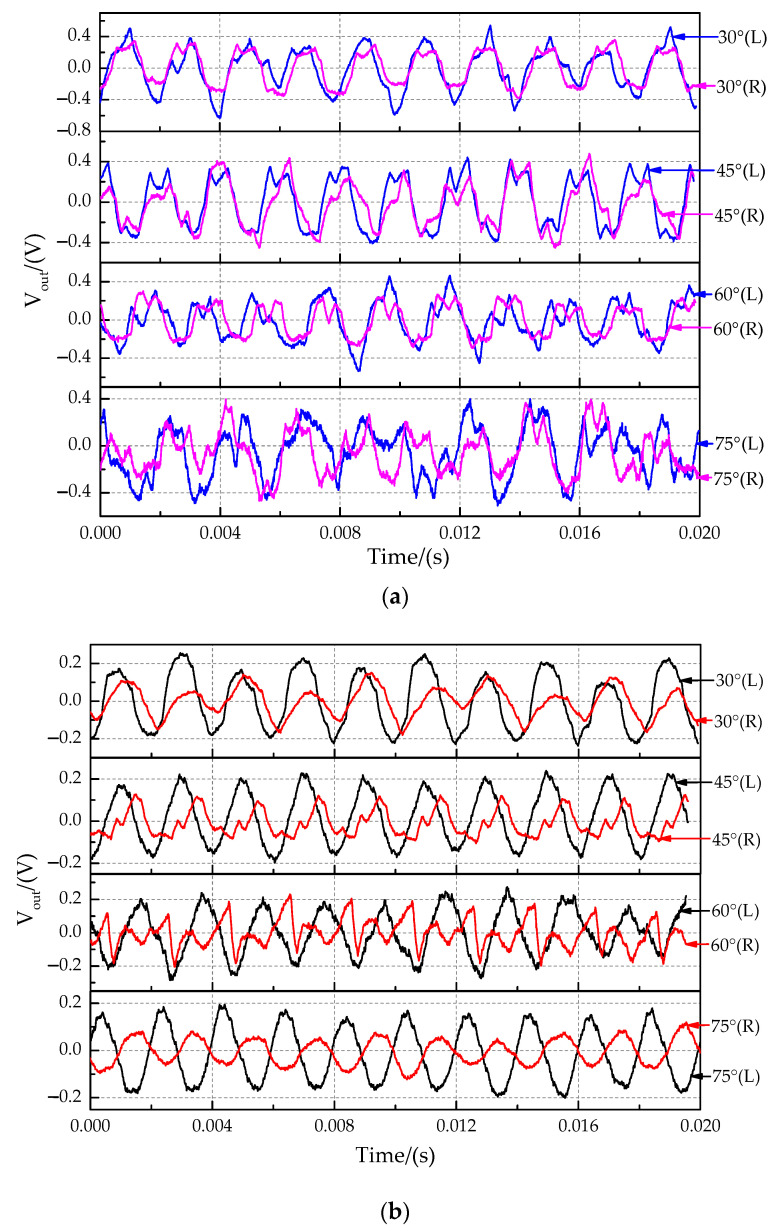
Time-domain characteristics of output signals at the sound source incident angle of 30°, 45°, 60° and 75°. (**a**) Non-coupling structure. (**b**) Coupling structure.

**Figure 17:**
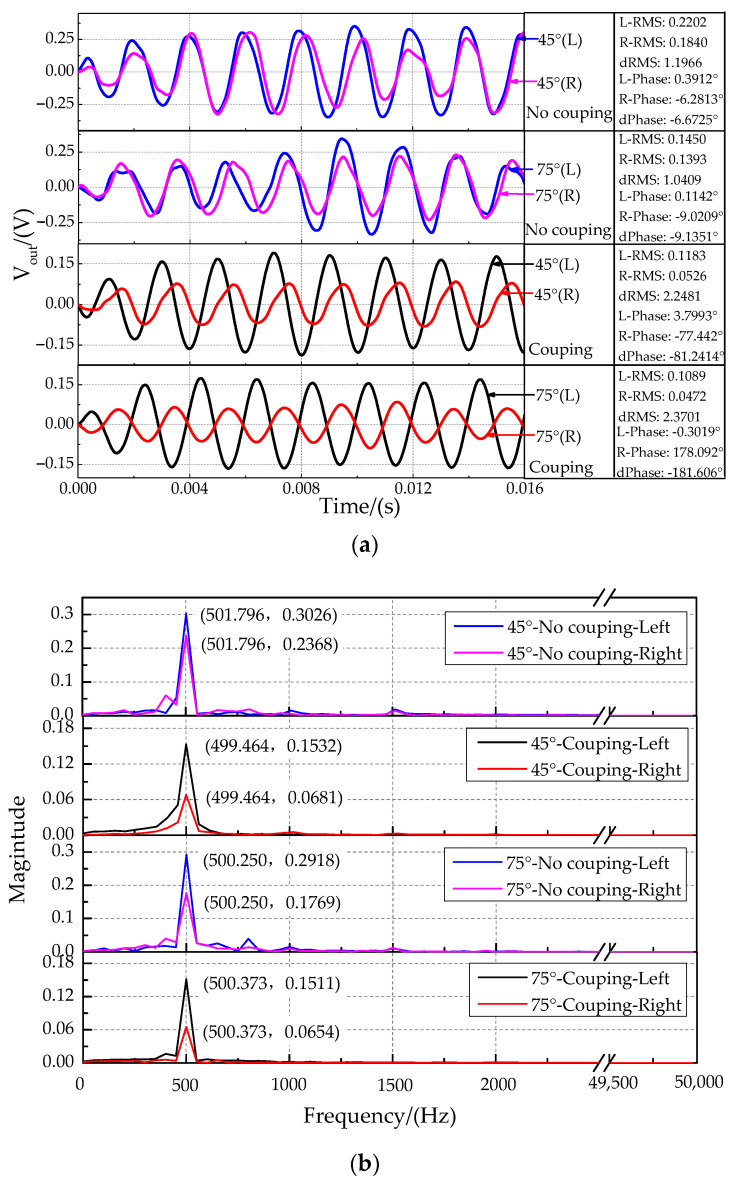
Characteristics at the sound source incident angle of 45°and 75°. (**a**) The time-domain characteristics. (**b**) The frequency-domain characteristics.

The time-domain characteristics of the output signals at sound source incident angle of 30°, 45°, 60°, and 75° are shown in Figure 16. Because the measured displacement is particularly small and affected by the external environmental and sensor resolution, the output signal has some distortion in Figure 16. The time-domain characteristics for the non-coupling structure (Figure 16a) and the coupling structure (Figure 16b) in this experiment showed that the phase of the right signal (far sensors) was delayed, and the amplitude of the right signal was suppressed compared with those of the left signal (near sensors). The time delay characteristic between the two signals was analyzed by comparing the phase difference of the fitted curve (Figure 17). The band-pass filter fitting method was used. The band-pass filter processing method filters out the high and low-frequency signals and retains the intermediate frequency signal, which is convenient to solve the signal amplitude and phase. The magnitude difference increases significantly.

**Figure 18:**
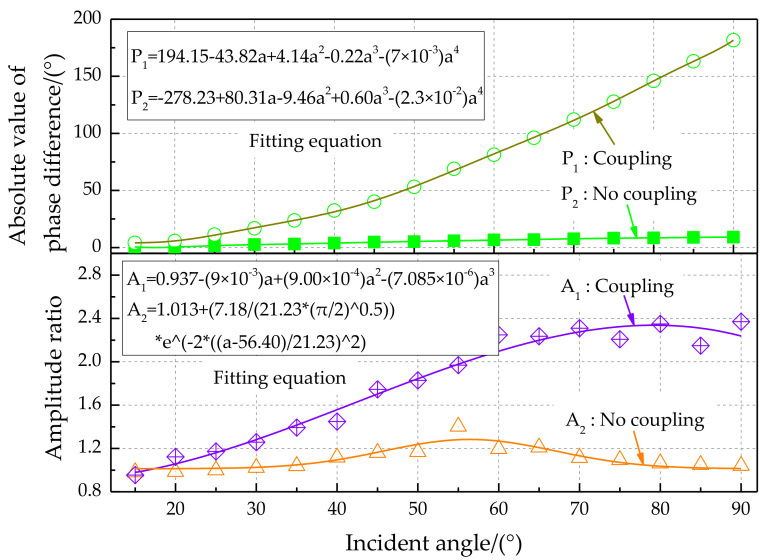
Phase difference and amplitude ratio characteristics for different incident angles of sound sources.

The phase difference amplification characteristics for different incident angles of sound sources are shown in Figure 18, top. Experimental results show that the absolute value of the phase difference value increases with increasing incident angle. The phase difference for the bionic coupling structure is significantly enlarged compared with the non-coupling structure. The change rate of the phase difference is approximately 2.5 (phase difference°)/(incident angle of sound source°) for the coupled acoustically sensitive structure. The theoretical resolution of the incident angle of the sound source can reach 2° at a phase difference recognition rate of 5°. The amplitude ratio characteristics for different incident angles of sound sources are shown in Figure 18, bottom. The amplitude ratio characteristics for the bionic coupling structure are significantly enlarged relative to those of the non-coupling structure. The third-order polynomial fitting method was used. Polynomial fitting uses a polynomial expansion to fit all observation points in a small analysis area containing several analysis grid points to obtain an objective analysis field, which is convenient for exploring the input–output relationship.

#### 4.2.2. Analysis of the Sound Source Frequency Characteristic

Second, the acoustic sensitivity characteristics at the sound source incidence angle of 45° and frequencies of 200 Hz, 500 Hz, 750 Hz, 1000 Hz, and 2000 Hz for the bionic coupling structure were tested. The remaining test conditions were the same as before.

**Figure 19:**
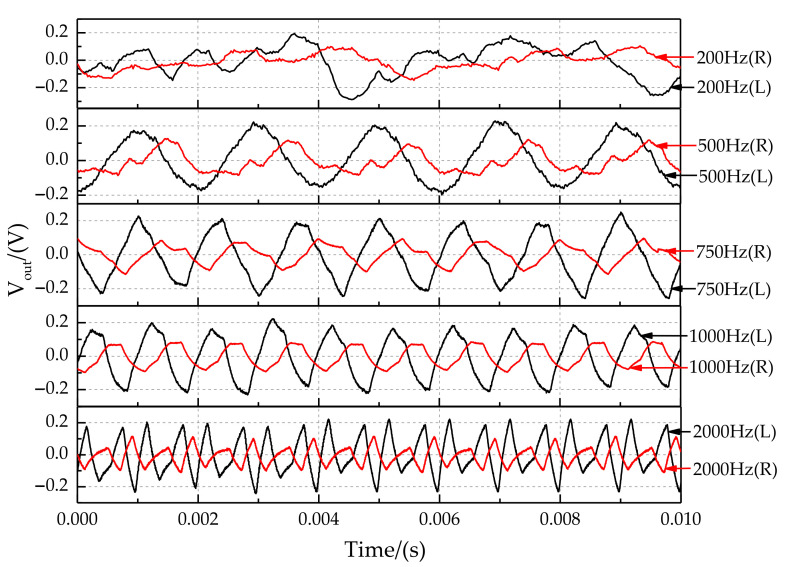
Characteristics of output signals at the sound source frequency of 200 Hz, 500 Hz, 750 Hz, 1000 Hz, and 2000 Hz.

The output signals at sound source frequencies of 200 Hz, 500 Hz, 750 Hz, 1000 Hz, and 2000 Hz are shown in Figure 19. The maximum sampling frequency of the laser sensor is 5000 Hz, so the signal was distorted when the frequency was 2000 Hz. In addition, experimental results show that the signal was doped with great noise when the sampling frequency was 200 Hz. In particular, the sound source signal’s recognition ability is lost when the frequency is lower than 200 Hz. At a sound source frequency of 200 Hz, the sound wavelength is 1.7 m. The distance between the coupled sensors is much smaller than 1/2 wavelength, so the coupling structure greatly increases the directional resolution of the low-frequency sound source. The optimal identification frequency range of the sound source for the coupling device is 300 Hz and 1500 Hz. It is in the middle and low-frequency band for the sound source.

**Figure 20:**
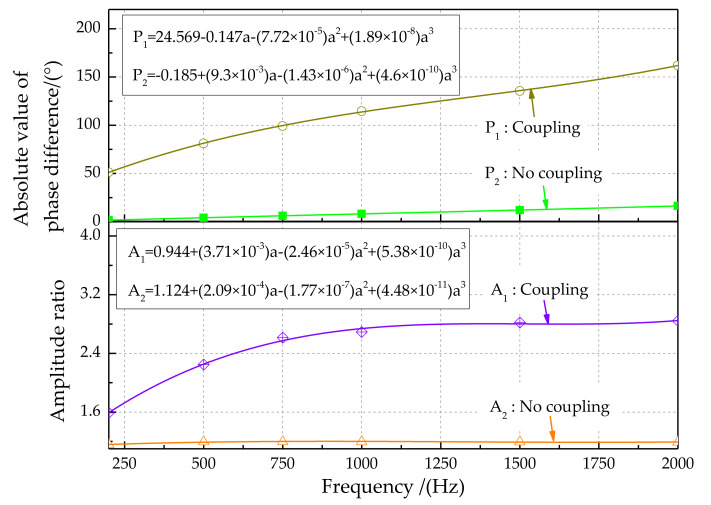
Phase difference and amplitude ratio characteristics of output signals for different sound source frequencies.

The characteristics of the output signals for different sound source frequencies are shown in Figure 20. The third-order polynomial fitting method was used to analyze signal characteristics. Experimental results show that the magnitude of the amplitude is greatly reduced. On the opposite side of the sound source, this phenomenon is more prominent. The phase difference characteristic (Figure 20, top) shows that the phase difference between the left and right signals increases with increasing sound source frequency, from 52.885° at 200 Hz to 145.596° at 2000 Hz. In addition, the phase difference between the left and right signals for the coupling structure is greater than that for the non-coupling structure. The amplitude ratio characteristic (Figure 20, bottom) shows that the amplitude ratio gradually increases with increasing sound source frequency. In addition, the sound source signal amplitude amplification effect for the bionic coupling structure is better. Therefore, the coupling device has a significant amplifying effect in terms of phase difference detection.

### 4.3. Recognition Experiment of the Direction Angle

The incident angle of the sound source was set as 36°, 42°, 48°, and 54° at the sound source frequency of 500 Hz, respectively, and the displacement response of the measuring point was measured. The calculated direction angle of the sound source was obtained by the identification methods of phase difference amplification and amplitude ratio amplification. A comparison of the calculated value and the actual value is shown in Table 2.

## 5. Conclusions

In conclusion, inspired by the super-acute ear of *O. ochracea*, the mechanical coupling principle was successfully applied to measuring the incident angle in a two-dimensional plane. A biomimetic study on the auditory localization mechanism of *O. ochracea* was performed to improve the localization ability of small acoustic systems. An amplifying localization mechanism of small differences was analyzed by studying acoustic excitation from the coupling structure to the ears. A method of estimating the direction angle of the sound source was proposed according to the amplitude difference and the phase difference of the system response based on the structural and mechanic model of the intermembrane bridge of *O. ochracea*. In addition, the simulation of the nonlinear characteristics of the structural model was performed by modal decomposition. We also present a microscale implementation of an acoustic localization device inspired by the auditory organ of the parasitic *O. ochracea*. The device consists of a pair of circular membranes coupled together with an elastic beam. The coupling serves to amplify the difference in magnitude and phase between the two membranes’ responses as the incident angle of the sound changes, allowing directional information to be deduced from the coupled device response. Experimental research on sound source localization microsensors based on intermembrane bridge bionic structures was executed to verify the simulation reliability. The simulation results show that the system model presented in this paper could effectively simulate the sound source localization mechanism of *O. ochracea*. The intermembrane bridge structure can effectively amplify the small phase difference and amplitude difference caused by the sound incident angle used in tiny sound detectors, which improves the sound source localization and directional weak acoustic signal acquisition of sound detectors. Experimental results show that the output signal’s shape is closer to a sine wave for the coupling structure. The recognition rate of the phase difference and amplitude ratio was greatly improved. The theoretical resolution of the incident angle of the sound source can reach 2° at a phase difference recognition rate of 5°. The sound source’s optimal identification frequency range for the coupling device based on the intermembrane bridge bionic structure is 300 Hz to 1500 Hz. Therefore, fly ear-inspired acoustic sensors offer great potential for developing small portable medium- and low-frequency sound source localization systems.

## Figures and Tables

**Table 1:** Structural parameters of the established inter-membrane bridge structure.

	Membrane	Bio-Bridge	Pivot 1	Pivot 2
**Equivalent Elastic modulus/(MPa)**	4000	97,000	2590	2880
**Poisson ratio**	0.3	0.32	0.3	0.3
**Density/(kg/mm^3^)**	1.38 × 10^−9^	8.65 × 10^−9^	1.55 × 10^−9^	1.55 × 10^−9^
**Size/(mm)**	Φ7 × 0.005	11 × 2 × 0.05	0.2 × 0.2 × 0.2	2 × 0.2 × 0.2
**Stiffness damping/(N∙s/mm)**			1.15 × 10^−8^	2.88 × 10^−8^

**Table 2:** Comparison of the calculated value and the actual value.

Actual Value	Calculated Value by Phase Difference Amplification Identification	Maximum Error	Calculated Value by Amplitude Ratio Amplification Identification	Maximum Error
36°	42.209°	+6.209°	39.177°	+3.177°
42°	36.413°	−5.587°	44.185°	+2.185°
48°	44.561°	−3.439°	43.204°	−4.796°
54°	49.339°	−4.661°	51.460°	−2.540°
**Mean of Absolute Value of Error**		4.974°		3.1745°

## Data Availability

Not applicable.

## Abbreviations

*O. ochracea*	*Ormia ochracea*
PET	Polyester
OSC	Oscilloscope
DAQ	Data acquisition
RMS	Root-mean-square
De	Decibel
Di	Distance from sound source
DOF	Degrees of freedom
